# Crack initiation and propagation in sweet cherry skin: A simple chain reaction causes the crack to ‘run’

**DOI:** 10.1371/journal.pone.0219794

**Published:** 2019-07-31

**Authors:** Christine Schumann, Andreas Winkler, Martin Brüggenwirth, Kevin Köpcke, Moritz Knoche

**Affiliations:** Institute for Horticultural Production Systems, Leibniz-University Hannover, Hannover, Germany; Universidade do Minho, PORTUGAL

## Abstract

Rain cracking severely affects the commercial production of many fleshy-fruit species, including of sweet cherries. The objectives were to investigate how the gaping macroscopic cracks (macrocracks) of a rain-cracked fruit can develop from microscopic cracks in the cuticle (microcracks). Incubating fruit in deionized water is well known to cause significant macrocracking. We found that after a lag phase of 2 h, the numbers and lengths of macrocracks increased. Macrocrack number approached an asymptote at 12 h, whereas macrocrack length continued to increase. The rate of macrocrack propagation (extension at the crack tip) was initially 10.8 mm h^-1^ but then decreased to a near-constant 0.5 mm h^-1^. Light microscopy revealed three characteristic zones along a developing macrocrack. In zone I (ahead of the crack), the cuticle was intact, the epidermal cells were unbroken and their cell walls were thin. In zone II, the cuticle was fractured, the first epidermal cells died and their cell walls began to thicken (swell). In zone III, most epidermal cells had died, their cell walls were swollen and cell:cell separation began along the middle lamellae. The thickness of the anticlinal epidermal cell walls and the percentage of intact living cells along a crack were closely and negatively related. Cracks were stained by calcofluor white, but there was no binding of monoclonal antibodies (mAbs) specific for hemicelluloses (LM11, LM21, LM25). Strong binding was obtained with the anti-homogalacturonan mAb (LM19), indicating the presence of unesterified homogalacturonans on the crack surface. We conclude that macrocrack propagation is related to cell death and to cell wall swelling. Cell wall swelling weakens the cell:cell adhesion between neighbouring epidermal cells, which separate along their middle lamellae. The skin macrocrack propagates like a ‘run’ in a fine, knitted fabric.

## Introduction

When it rains near harvest, rain cracking can reduce both the yield and the quality of many species of fleshy fruit. Sweet cherry, grape and tomato are the most significant commercial crops thus affected–significant both because of the large scale of these industries and also because of the extreme susceptibility of these species to damage [1–4].

In recent years, and working mainly with sweet cherry, significant progress has been made in understanding the mechanistic basis of rain cracking–deep macrocracks that breach the skin and run deep into the flesh. Such macrocracks expose the interior of the fruit to rapid and catastrophic degradation through the combined ravages of drying, microbial invasion and sugar-seeking insects.

Till now, studies on rain cracking have focused on cuticle deposition [5], microcracking of the cuticle [6], the analysis of stress and strain in the skin [7, 8], the mechanical properties of the skin [9, 10] and the mechanisms/pathways of water movement through the skin [11, 12] and through the vascular system from the parent tree/vine [13].

Although it is thought that macrocracks develop from microcracks [6, 14]. Surprisingly little information is available on the final step of the macrocracking process–the initiation and propagation of these visible skin cracks. Considering the importance of fruit macrocracking, the lack of information on this putative link with microcracking is surprising.

In engineering, the analysis of fracture surfaces provides important clues as to the reason for the mechanical failure. Indeed, fractography has become a forensic discipline within materials science, where fracture surfaces are studied to identify the causes of failure in engineering structures, e.g. in failed buildings, bridges, airframes, etc. [15–17]. Fractography has also found a place in the study of animal structures, where, for example, it is used to develop and evaluate theoretical models of crack propagation in bones [18]. Here, we apply some of these engineering principles to plant structures and, in particular, to the skin of sweet cherry.

Fruit skins suffer of two different failure modes [19]. A fruit skin can fracture due to failure of the skin cell wall–here the failure line runs **across** the cell wall (lysigeny) and the cell contents are lost. Alternatively, a fruit skin can fracture due to the failure of cell:cell adhesion–here the failure line runs **along** the cell wall (schizogeny) and adjacent cells separate from one another along their middle lamellae and each cell remains intact [20].

Schizogenous skin failure is characteristic of sweet cherry fruit skins in the field and this failure is usually associated with rainfall. Schizogenous skin failure also occurs in the laboratory when a sweet cherry fruit is incubated in deionized water [21]. The fraction of cells failing along cell walls (schizogenously) was closely and positively related to the extent of cell wall swelling [22]. Although the detailed mechanism of schizogeny in this context has yet to be determined, the pectins of the middle lamellae are thought likely candidates for the associated cell wall swelling [23, 24]. We would thus expect pectins to be exposed on the broken surfaces of a developing macrocrack.

Immunolabeling of cell wall epitopes has proved a useful technique for obtaining detailed information on the spatial distribution of cell wall carbohydrates in a plant tissue [25, 26]. With immunolabeling, a primary monoclonal antibody from the LM series (generated in rat) that is specific for a particular cell wall epitope, is bound to the tissue. Next, a secondary antibody (anti rat) carrying a fluorescent tracer is applied that binds to the primary cell wall specific antibody. This antibody can then be detected by fluorescence light microscopy. Immunolabeling has been used to identify constituents of cell walls that are involved in cell to cell adhesion [27]. Usually, the tissue is fixed to prepare thin-sections for light microscopy. However, in principle, the technique should also be able to be used with a fresh tissue, for example to identify cell wall constituents exposed on the facture surface of macrocracked cherry fruit. Nevertheless, its use with a fresh tissue poses some challenges. First, tissue sectioning must be avoided because it is inevitably accompanied with stress and strain relaxation [7]. Second, the use of antibodies requires very small volumes of reaction solution and, hence, excludes the incubation and submersion of a whole cherry fruit. Third, artifacts resulting from uptake of water by the fruit, from the reaction solution, must be excluded as a factor. These difficulties may be avoided. The first by using intact fruit (no cutting), the second by restricting the reaction solution to a tiny portion of the fruit surface and the third by using an osmotically-buffered isotonic reaction solution.

The purpose of our study was: 1) to characterize the initiation of macrocracks (macroscopic cracks) in intact cherry fruit, 2) to determine the relationship between macrocrack initiation and cell wall swelling and 3) to identify the cell wall constituents exposed on the surface of a propagating macrocrack. We used sweet cherry fruit as a model system because there is a large body of published information on this economically important fruit crop.

## Materials and methods

### Plant material

Mature fruit of sweet cherry (*Prunus avium* L.) were sampled from trees of the cultivars Adriana, Burlat, Early Korvic, Gill Peck, Hedelfinger, Kordia, Regina, Sam, Samba and Sweetheart. All cultivars were grafted on ‘Gisela 5’ rootstocks (*P*. *cerasus* L. x *P*. *canescens* Bois). The trees were cultivated in a greenhouse or under a rain shelter at the horticultural research station in Ruthe (52.2N, 9.8E) or in the open field at an experimental orchard in Hannover (52.4N, 9.7E) according to current regulations for integrated fruit production. Fruit were picked at commercial maturity based on color, selected for freedom from defects and for uniformity based on color and size. They were used for the experiments on the same day.

### Macrocracking assays

The position of macrocracks on the fruit surface and their propagation were recorded by incubating cultivars Adriana, Early Korvic, Gill Peck and Hedelfinger fruit in deionized water in polyethylene (PE) boxes placed on a light box in a temperature controlled room at 22°C. The pedicel was cut to a length of about 5 mm. Fruit were held in position in the PE box using silicone rubber (RTV 3140; Dow Corning, Midland, MI). After allowing the silicone rubber to cure for 30 min, the PE boxes were positioned under a custom multiple camera stand, equipped with six digital cameras (WG-20; Ricoh, Tokyo, Japan). Each camera viewed six fruit simultaneously. Cameras were set in time-lapse mode and images were recorded every 2 h. Before initiation of the cracking assay, the camera setup was calibrated. This setup allowed monitoring of the time course of macrocrack propagation in the regions of the pedicel cavity, the cheek, the suture, the left and right shoulders and the stylar scar.The number of fruit replicates was six. A detailed short term time course was established by analyzing thirteen macrocracks at 10 min intervals. The macrocracks selected for this analysis fulfilled the following requirements: They progressed without branching and stayed within a particular region. The region monitored remained free of other macrocracks.

**Fig 1:**
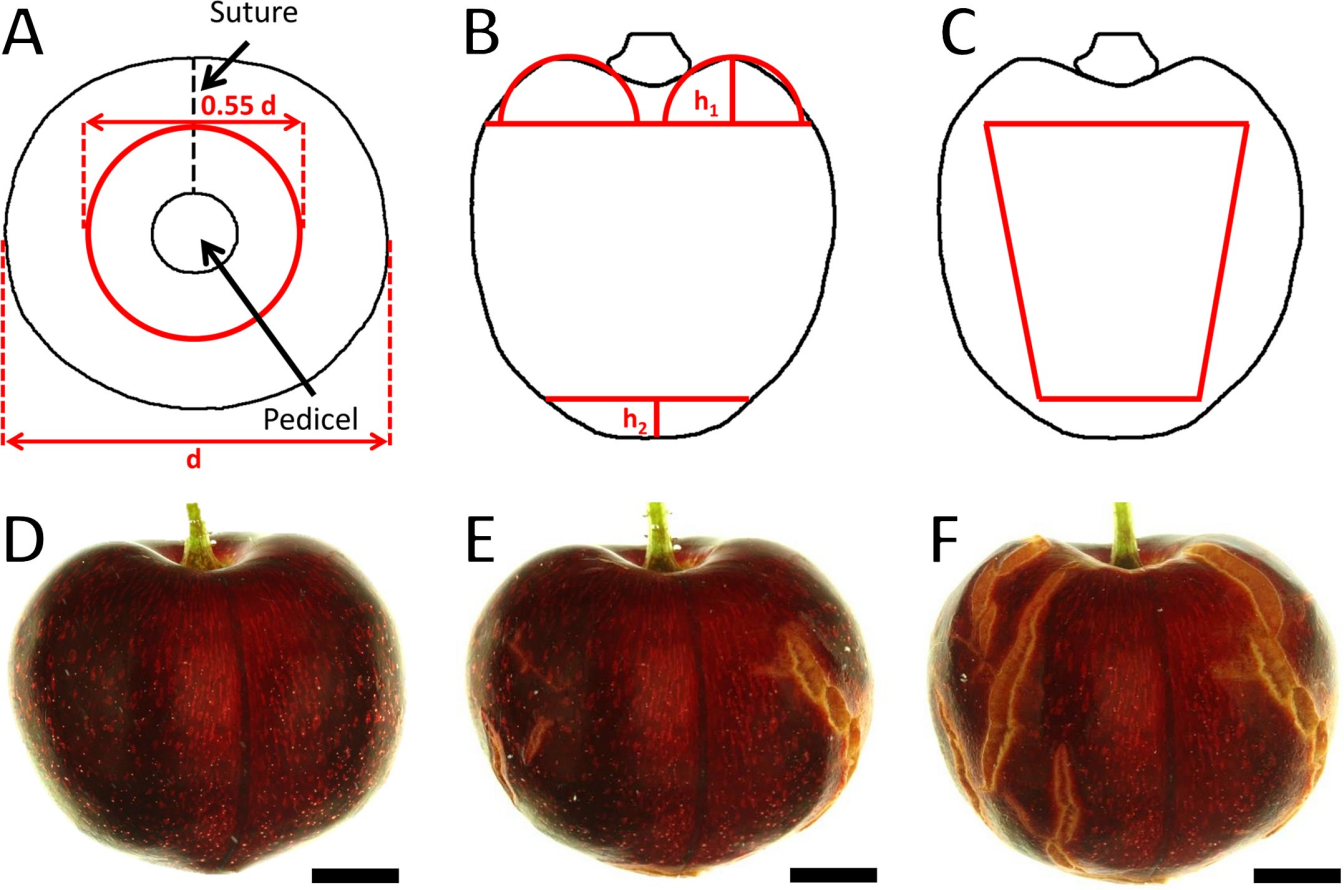
Schematic illustrating the procedure used to quantify crack number and crack length on the six orthogonal regions of the fruit surface. A) Top view of a fruit with receptacle and pedicel in the center of a circle. B) Front view of a fruit illustrating the pedicel cavity and stylar scar regions. The pedicel cavity region was approximated by a circle with 0.55 times the diameter of the fruit and a (half) torus having a radius defined as height *h*_*1*_. The stylar scar region was approximated by the cap of a sphere of height *h*_*2*_. C) Side view of a fruit with four trapezoids (only one is visible) representing the cheek, the suture and the two shoulders of a fruit. The trapezoids were drawn such that their heights were delineated by the base of the torus marking the pedicel cavity region, and their bases by the cap delineating the stylar scar region. The vertical margins of each trapezoid were delineated by the respective width of the fruit from which a 10% boundary was subtracted to minimize errors due to curvature of the surface. D-F) Representative images taken from a video clip at 0 h (D), 13 h (E) and 24 h (F) of the suture region of a ‘Regina’ sweet cherry when incubated in deionized water. Scale bar = 5 mm.

So that no crack was counted twice and errors due to surface curvature remained below 10%, the fruit surface was partitioned into six orthogonal windows (1…6) for data analysis. To delineate the pedicel cavity 1) and stylar-scar 2) regions, a circle of 0.55 times the diameter *d* was drawn about the fruit long axis (Fig 1A). To delineate the cheek 3) and the suture 4) and the two shoulders 5–6), four trapezoids were used. The top half of a torus of height h_1_ was positioned in the pedicel cavity region and used to define the upper margins of the four trapezoids (Fig 1B) while their lower margins were defined by the cap of a sphere. The height h_2_ of the cap was calculated from the maximum diameter *d* of the fruit:

$$h_{2}=\frac{d}{2}-\sqrt{(\frac{d}{2}{)}^{2}-{0.55*d}^{2}}.$$

A 10% safety distance was subtracted from each of the four sides and the points were connected to form four trapezoids (Fig 1C).

The numbers and lengths of macrocracks and the cumulative crack lengths within each of the six windows were measured using image analysis (cellSens Dimension 1.7.1; Olympus, Hamburg, Germany).

### Light microscopy

Mature fruit, free from visible defects, were incubated in deionized water at 22°C for 8 to 22 h to induce macrocracking. Epidermal segments were excised by tangential cuts using a razor blade. Segments were promptly mounted on a glass slide, transferred to the stage of a light microscope (BX-60; Olympus, Hamburg, Germany; Axioplan; Carl Zeiss Microscopy, Jena, Germany) and covered with silicone oil to prevent water uptake/loss. Subsequently, segments were inspected for microcracks at ×40 in transmitted white light. Calibrated light micrographs of tips of cracks were taken using a digital camera (DP71 and DP73; Olympus, Hamburg, Germany). Thicknesses of anticlinal epidermal cell walls were measured along a developing crack in zones I, II and III using image analysis (CellSens; Olympus, Hamburg, Germany). The three crack zones are defined as proposed by [22]. Briefly, zone I represents the healthy epidermal tissue ahead of a crack, characterized by an intact cuticle. In zone II a microcrack has developed in the cuticle. In zone III the microcrack has extended through the cuticle deep into the underlying epidermis, hypodermis and outer cortex. The cracking in zone II is microcracking, these fine cracks do not extend any deeper than the base of the cuticle and are barely visible to the naked eye [6]. Zone III cracks are clearly visible to the naked eye, macrocracks, and extend deep into epidermis and hypodermis and later into the flesh, sometimes right down to the pit.

The time course of swelling of anticlinal epidermal cell walls and the percentages of intact vital and dead epidermal cells along a developing macrocrack were determined on the cheek of ʹReginaʹ. Dead cells were identified by their brownish coagulated cytoplasm and the loss of anthocyanin from the vacuole.

Swelling of epidermal cell walls was also compared across cultivars. Fruit of cultivars Adriana, Kordia, Regina, Sam and Sweetheart were incubated in deionized water for 18–23 h at room temperature (22°C) before cell wall swelling was assessed.

Subsequently, cell wall swelling in the cheek, the pedicel cavity and the stylar scar regions of ‘Regina’ fruit was recorded and analyzed.

Fruit of ‘Regina’ sweet cherry were incubated in 70 mM malic acid for 20 h at 22°C and the effects on cell wall swelling and on the percentages of ruptured and intact epidermal cells were recorded. Malic acid at this concentration was used because 1) it corresponds to the typical concentration of this moiety in the expressed juice of mature sweet cherry fruit [28] and 2) because the release of juice (containing malic acid) from ruptured mesocarp cells is known to be a crucial factor in the propagation of macrocracks in mature sweet cherry fruit [29, 30]. Fruit incubated in deionized water served as controls.

### Immunolabeling of cell walls

The cell wall constituents exposed on the broken surface of cracks in mature fruit of cultivars Burlat and Samba sweet cherry were identified by immunolabeling of the exposed epitopes. The pedicel was removed and pedicel cavity, including receptacle and pedicel end, sealed with silicone rubber (3140 RTV Coating; Dow Corning, Midland, MI, USA). This procedure restricted water uptake to the fruit surface [31]. Sections of silicone rubber tubing (inner diameter 14 mm) were mounted in the stylar scar region of sweet cherry fruit and allowed to cure for 2 h. The sections of the tubing served as wells for solutions used in the labeling process. Fruit with tube sections attached were incubated in deionized water overnight. In the mornings, fruit were selected for immunolabeling which had cracks of comparable dimensions in the attached tube sections. The entire labeling procedure was carried out using fresh (i.e. non-fixed) tissue. We followed the multi-step protocol described by [26] and [27] with minor modifications. (I) Non-specific protein binding sites in the crack were blocked using 200 μl of isotonic phosphate-buffered saline (PBS) containing 3% (w/v) nonfat dry milk powder and held in the well for 30 min. Tonicity of the buffer was adjusted using glucose. (II) After removal of the blocking solution, the surface was thoroughly washed for 3 x 5 min using three changes of isotonic PBS to remove any remaining milk protein from the surface. (III) The following primary monoclonal antibodies (mAbs; PlantProbes, Leeds, UK) were prepared in a ten-fold dilution in isotonic PBS buffer at pH 7.0. The mAbs reacting with hemicelluloses were: LM11 (anti-xylan/arabinoxylan [32]), LM21 (anti-mannan [33]) and LM25 (anti-xyloglucan [34]). Those against pectins were: LM5 (anti-galactan [35]), LM6 (anti-arabinan [36]), LM7 (anti-homogalacturonan [37]), LM8 (anti-xylogalacturonan [38]), LM19 (anti-homogalacturonan [39]) and LM20 (anti-homogalacturonan [39]). The mAb 2F4 (anti-homogalacturonan [40]) was made up in 20 mM Tris(hydroxymethyl)methylamin buffer (TRIS-HCl) adjusted to pH 8.2. All primary mAbs from the LM series were anti rat. Only 2F4 was anti mouse. Solutions were applied to the wells on the fruit (200 μl each) and allowed to react for 1 h at room temperature (c. 22°C). Buffer solution containing no primary mAbs was applied to the control. The mAb 2F4 that identifies Ca^2+^ crosslinks in homogalacturonans was also applied in the presence of 0.5 mM CaCl_2_, as a second control. The second control was needed to demonstrate potential Ca^2+^ binding sites on the surface of a macrocrack. (IV) The solutions containing the primary antibodies were removed and the wells were carefully washed using isotonic PBS buffer. (V) The antibodies bound to the exposed cell wall epitopes were next labeled using a secondary antibody carrying a fluorescence marker (Alexa Fluor 488 anti rat for LM mAbs and Alexa Fluor 488 anti mouse for 2F4). The treatment solutions comprised a 200-fold dilution of the secondary antibody prepared in isotonic PBS buffer (200 μl per well). Fruit were incubated for 1.5 h at room temperature (22°C) in darkness in PE boxes at high humidity (maintained using wet filter paper). (VI) Fruit surfaces were rinsed twice with isotonic PBS (5 min each) and subsequently treated with 0.1% (w/w) calcofluor white (fluorescent brightener 28; Sigma-Aldrich Chemie, Munich, Germany) solution for 5 min to identify cellulosic cell walls exposed in the cracks. (VII) The calcofluor white solution was removed, the surface washed once with deionized water before 200 μl of a PBS-based antifadent solution (Citifluor AF3; Science services, Munich, Germany) was applied. Preliminary experiments established that the antifadent solution prevented the decrease in signal intensity that occurred following the staining procedure. Finally, (VIII) the antifadent solution was removed and the surface in the well carefully blotted dry. The antibody-labeled fruit were viewed using a dissecting fluorescence microscope (MZ10F; filters GFP-plus 480–440 nm excitation, ≥510 nm emission; UV 360–440 mm excitation, ≥420 emission; Leica Microsystems, Wetzlar, Germany) at ×0.8, ×2, ×4 and ×8 equipped with epifluorescence. For comparing the bound antibodies, exposure times were held constant and set to 3, 8 and 15 s at ×0.8 and to 0.3 and 0.8 s at ×2, ×4 and ×8. For viewing calcofluor white stained specimens, exposure times were 1 and 3 s at ×0.8 and at 0.3 s at ×2, ×4 and ×8.

### Data analysis and terminology

Data are presented in scatter plots and tables as means ± standard errors of the means. Where not shown, error bars were smaller than data symbols. Data were analyzed by analysis of variance using the statistical software package SAS (version 9.1.3; SAS Institute, NC, USA).

We refer to the thickness of anticlinal epidermal cell walls as ‘cell wall thickness’ and to the increase in their thickness as ‘swelling’. Our thickness estimates comprise the sum of the thicknesses of the two cell walls of abutting epidermal cells, plus the pectin middle lamella between the two cells.

## Results

**Fig 2:**
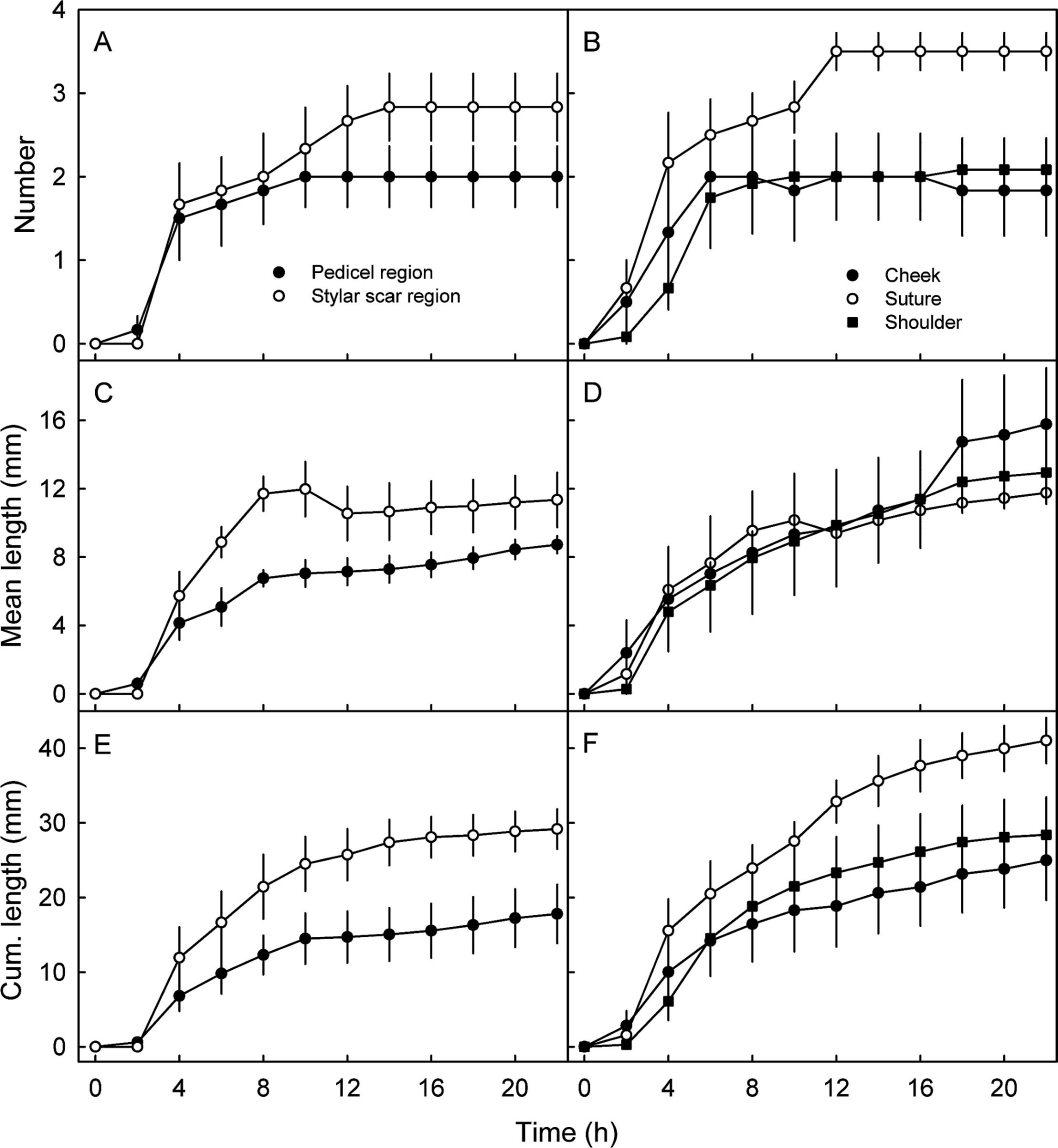
Time course of macroscopic cracking in different regions of the fruit surface of ‘Kordia’ sweet cherry. A,C,E. Numbers of macrocracks (A), average lengths of macrocracks (C), and cumulative length of macrocracks (E) in stylar scar and pedicel cavity regions. B,D,F. Number of macrocracks (B), average lengths of macrocracks (D) and cumulative lengths of macrocracks (F) in cheek, suture and shoulder regions. Fruit were incubated in deionized water to induce cracking.

Incubating mature fruit in deionized water caused significant macroscopic cracking (Fig 1D–1F). After a lag phase of about 2 h of incubation, crack number (Fig 2A and 2B), mean crack length (Fig 2C and 2D) and cumulative crack length per fruit all increased (Fig 2E and 2F). This pattern was consistent across all regions of the fruit surface and across all cultivars (Table 1).

**Table 1:** Number of cracks and cumulative length of cracks per fruit in various sweet cherry cultivars.

Cultivar	8 h			22 h		
	Cracks (No. per fruit)	Mean length (mm)	Cum. length (mm per fruit)	Cracks (No. per fruit)	Mean length (mm)	Cum. length (mm per fruit)
Adriana	-^a^	-	-	4.7 ± 1.0 b	8.7 ± 1.7 c	40.6 ± 10.3 b
Early Korvic	3.3 ± 0.3 b^b^	6.4 ± 0.6 b	21.4 ± 2.3 b	4.3 ± 0.7 b	8.9 ± 1.0 bc	38.6 ± 5.4 b
Gill Peck	2.3 ± 0.4 b	8.6 ± 1.2 b	20.0 ± 4.3 b	3.3 ± 0.8 b	12.7 ± 1.6 bc	42.3 ± 5.4 b
Hedelfinger	1.2 ± 0.5 b	9.6 ± 1.2 ab	11.2 ± 5.8 b	2.0 ± 0.6 b	15.7 ± 1.7 ab	31.5 ± 7.0 b
Kordia	7.7 ± 1.4 a	14.6 ± 1.0 a	111.8 ± 10.9 a	8.7 ± 1.0 a	19.6 ± 1.0 a	169.8 ± 11.3 a

Fruit was incubated in deionized water for up to 22 h to induce cracking.

^a^No cracked fruit.

^b^Mean separation within columns by Tukey’s Studentised range test (<5%).

Quantitatively, cracking was more severe in the stylar scar and the pedicel cavity regions compared in the cheek, suture or shoulder regions (Fig 2) and more severe in cv. ‘Kordia’ compared with all other cultivars (Fig 2, Table 1).

**Fig 3:**
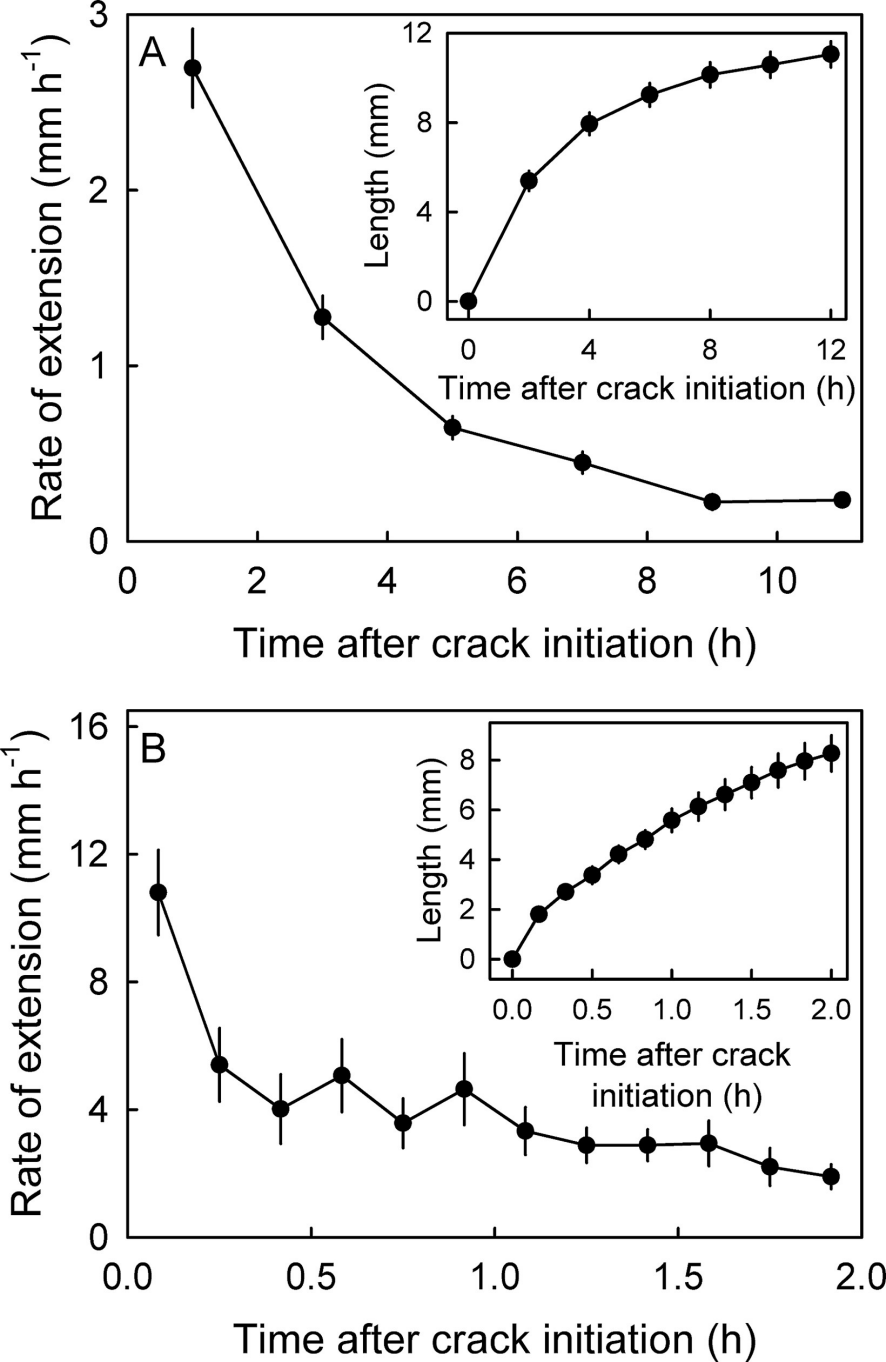
**Long term (A) and short term time courses (B) of change in rate of extension of macrocracks (main graphs) and in length of individual macrocracks (insets) in ‘Kordia’ sweet cherry.** Fruit were incubated in deionized water to induce cracking.

Averaged across regions, length of a macrocrack increased with time (Fig 3A, inset). The extension rate was most rapid immediately after crack initiation and then decreased (Fig 3A). A detailed short term time course revealed thatmaximum rates of on average 10.8 ± 1.3 mm h^-1^ (range 2.6 to 17.8 mm h^-1^) were measured within the first 10 min of macrocrack formation (Fig 3B). Thereafter, the rates of extension of macrocracks slowed to about 0.5 mm h^-1^ (Fig 3A)

**Fig 4:**
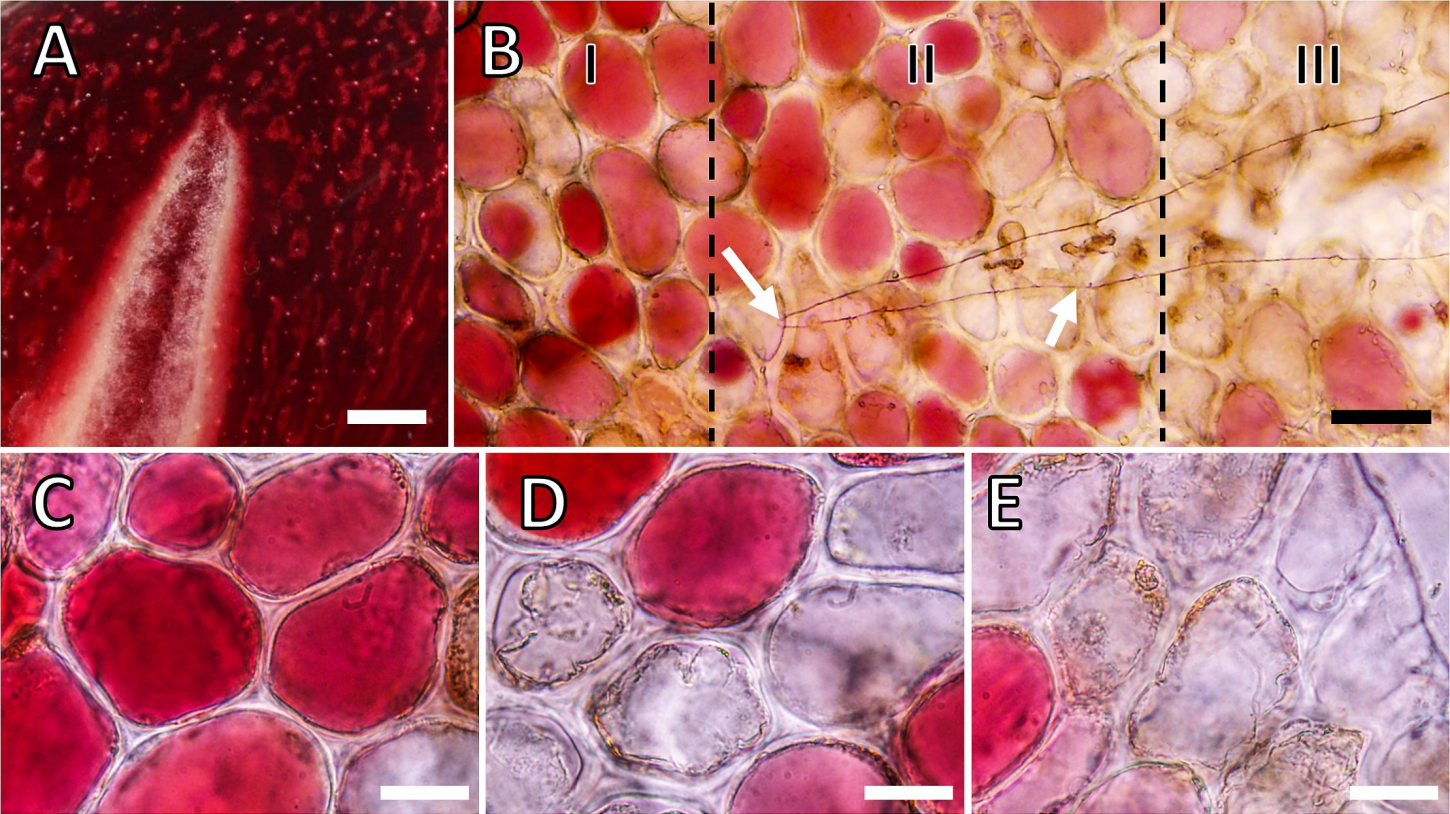
Micrographs of macroscopic cracks (macrocracks) and microscopic cracks (microcracks) in the skin of ‘Regina’ sweet cherry. A) Close up view of the tip of an extending macrocrack. B) Light micrograph of the tip of a macrocrack in the cuticle of the skin of a mature sweet cherry fruit depicting zones I, II and III of a developing macrocrack. C) Light micrograph of fruit skin in zone I. Zone I is the zone ahead of a developing crack with an intact cuticle and adherent, still-living, epidermal cells. D) Light micrograph of fruit skin in zone II. Zone II represents the tip of the microcrack (indicated by the white arrow) showing rupture of the cuticle. In zone II, the first separation of epidermal cells occurs, some of which are dead. E) Light micrograph of fruit skin in zone III. In zone III, the microcrack develops into a macrocack that extends deep into the epidermal and hypodermal cell layers and begins to gape. Essentially all the cells along a macrocrack are dead. Bars in A = 1 mm, B = 50 μm, C-E = 20 μm.

**Fig 5:**
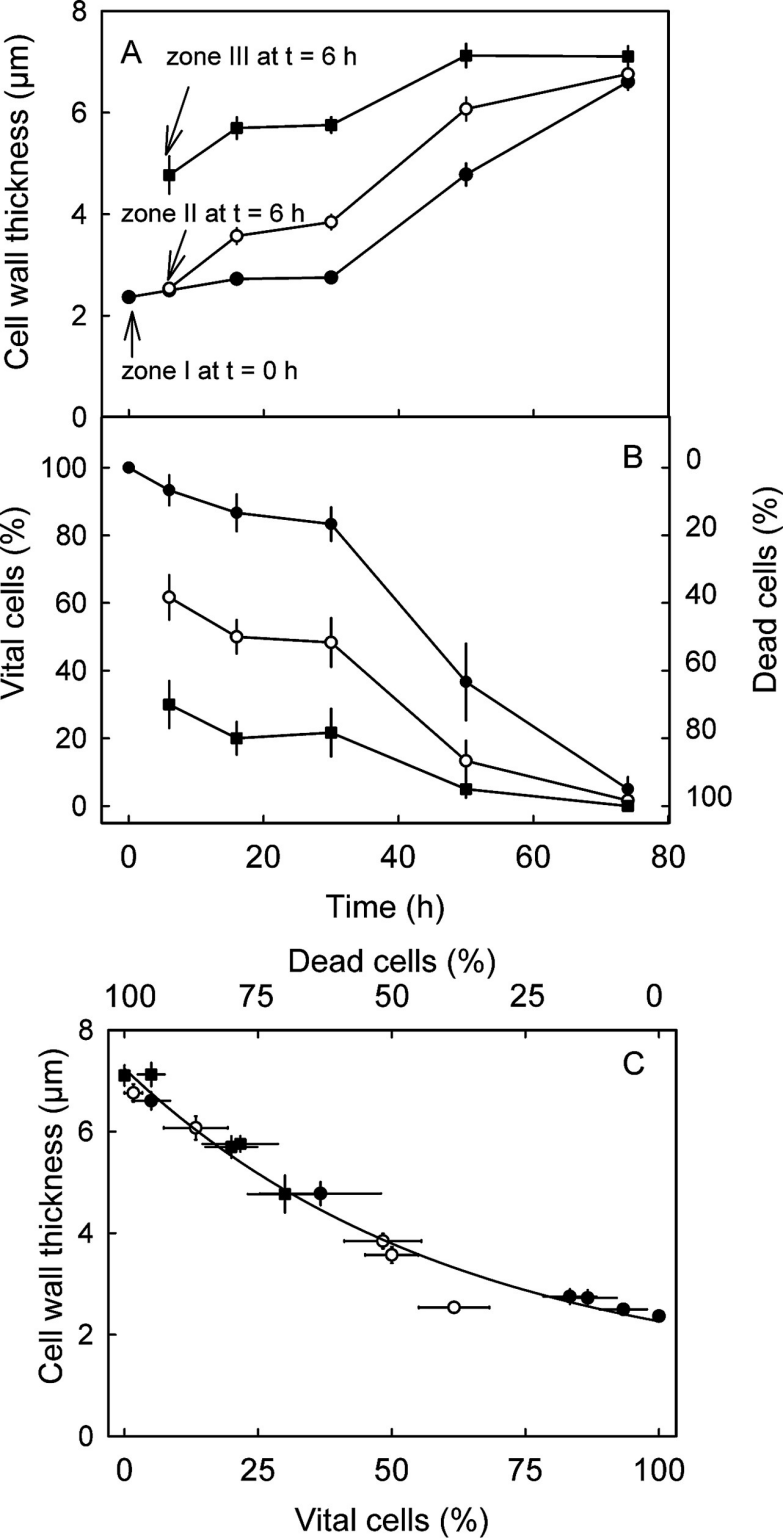
**Time course of cell wall swelling (A) and the proportions of living vs. dead cells (B) along a developing macrocrack. C) Relationship between cell wall thickness and percentage of living cells.** ‘Regina’ fruit were incubated in deionized water and removed from solution at different times to investigate the development of a skin macrocrack. Zone I represents the intact, healthy skin ahead of a macrocrack at time zero (t = 0 h), zone II the tip of a macrocrack at t = 6 h and zone III a macrocrack where gaping has begun. The macrocrack is now bordered by dead cells.

Light microscopy of the tips of developing macrocracks revealed the three characteristic zones along a crack (Fig 4A and 4B). In zone I, ahead of the crack, epidermal cells were closely adherent, their anticlinal walls were thin (non-swollen) and they were intact (Fig 4B, Fig 5A). In zone II, the cuticle had fractured, the epidermal cells just beneath the microcrack were still adherent and many had died as indexed by the presence of coagulated cytoplasm and loss of anthocyanin from their vacuoles. Finally, in zone III, the microcrack had widened and deepened into the epidermis and hypodermis of the skin and into flesh to form a gaping macrocrack. The epidermal cell walls were swollen and had separated from one another (predominately along their anticlinal walls) and nearly all were dead.

Monitoring cell wall swelling during crack development indicates that–once a microcrack has been initiated–the thickness of the anticlinal cell walls increases with time (Fig 5A). The increase in cell wall thickness in zone III exceeded that in zone II, which exceeded that in zone I. By 72 h, there was essentially no difference in cell wall thickness between the three former zones of a crack. As crack development proceeded, the percentage of living cells decreased. Again, the proportion of living cells was least in zone III, and greater in zone II, while all appeared to be alive in zone I (Fig 5B). Differences in cell wall swelling and cell death between the former zones diminished with time and as the microcrack deepened into a gaping macrocrack. There was a close negative relationship between the thickness of anticlinal cell walls and the percentage of still-living cells along a macrocrack (Fig 5C).

Swelling of anticlinal epidermal cell walls during macrocrack formation was observed in all cultivars investigated (Table 2). However, initial cell wall thickness (zone I, ahead of a macrocrack) and the extent of cell wall swelling during macrocracking, did depend on cultivar. ‘Regina’ and ‘Sam’ sweet cherry had the thickest cell walls, and ‘Adriana’, ‘Kordia’ and ‘Sweetheart’ had the thinnest. The increase in cell wall thickness upon macrocracking was largest in ‘Regina’ and Sweetheart, and least in ‘Kordia’, ‘Sam’ and ‘Adriana’.

**Table 2:** Cell wall thickness in different zones of a developing macrocrack in the fruit skin.

Cultivar	Cell wall thickness (μm)			
	Zone I	Zone II	Zone III	Zone III–zone I^b^
Adriana	2.8 ± 0.1aA^a^	2.9 ± 0.1aA	3.7 ± 0.2bA	0.9 ± 0.2
Kordia	2.8 ± 0.2aA	4.8 ± 0.2bB	5.6 ± 0.3cB	2.7 ± 0.3
Regina	4.0 ± 0.2aB	5.8 ± 0.3bC	7.5 ± 0.4cC	3.5 ± 0.4
Sam	3.9 ± 0.2aB	4.6 ± 0.3abB	6.0 ± 0.3bB	2.1 ± 0.3
Sweetheart	2.7 ± 0.1aA	4.5 ± 0.2bB	5.9 ± 0.3cB	3.2 ± 0.3
Grand Mean	3.3 ± 0.1	4.5 ± 0.2	5.7 ± 0.3	

Fruit were incubated in deionized water to induce macrocracking. Zone I is just ahead of a developing macrocrack. Zone II is where the cuticle has cracked (microcracked) but not the underlying cellular layers. Zone III is where cell separation has begun and a macrocrack is clearly to be seen.

^a^Means within rows followed by the same lowercase letter and those within columns followed by the same uppercase letter are not significantly different (<5%).

^b^Cell wall thickness in zone I subtracted from cell wall thickness in zone III.

Swelling of cell walls did not differ significantly between regions of a fruit within a cultivar (Table 3).

**Table 3:** Cell wall thickness in different zones of a macrocrack in the cheek, pedicel cavity and stylar scar regions of ‘Regina’ sweet cherry.

Region	Cell wall thickness (μm)		
	Zone I	Zone II	Zone III
Pedicel cavity	4.2 ± 0.3	4.8 ± 0.3	6.4 ± 0.6
Cheek	3.3 ± 0.4	4.4 ± 0.6	6.4 ± 0.8
Stylar scar	4.2 ± 0.2	5.7 ± 0.3	6.4 ± 0.2
Grand Mean	4.0 ± 0.1 a ^a^	5.1 ± 0.2 b	6.4 ± 0.2 c

Fruit were incubated in deionized water to induce cracking. Zone I is ahead of a macrocrack, zone II is where the cuticle has cracked (microcracked) and zone III is where cell separation has begun (a macrocrack is forming).

^a^Two-factorial analysis of variance revealed a significant main effect for ‘zone’, the main effect for ‘region’ and for the interaction ‘region x zone’ were not significant. Means followed by the same letter do not differ significantly (<5%).

Malic acid at a concentration typical for juice of sweet cherry dramatically increased swelling of cell walls (Table 4). In the presence of malic acid, epidermal cell walls were consistently thicker in all zones than in those of water treated controls. It is interesting that, compared to the water controls, in the malic acid treated skins, the cell walls were about twice as thick and a large proportion of the cells was dead, even in zone I, ahead of the macrocrack. In zone 1 of the control, cell walls remained thin and all cells appeared alive.

**Table 4:** Effect of malic acid on cell wall thickness in different zones of a developing skin macrocrack of ‘Regina’ sweet cherry.

Regions	Cell wall thickness (μm)		
	Zone I	Zone II	Zone III
Control	3.3 ± 0.2 a ^a^	4.5 ± 0.2 a	6.0 ± 0.2 a
Malic acid	7.7 ± 0.3 b	8.0 ± 0.2 b	8.7 ± 0.3 b

To induce macrocracking, fruit were incubated for 20 h in deionized water (control) or in 70 mM malic acid (treatment). Zone I lies ahead of a macrocrack, zone II is where the cuticle has cracked (microcracked) and zone III is where cell separation has begun (a macrocrack is forming).

^a^Mean separation within columns by Tukey’s Studentised range test (<5%).

**Fig 6:**
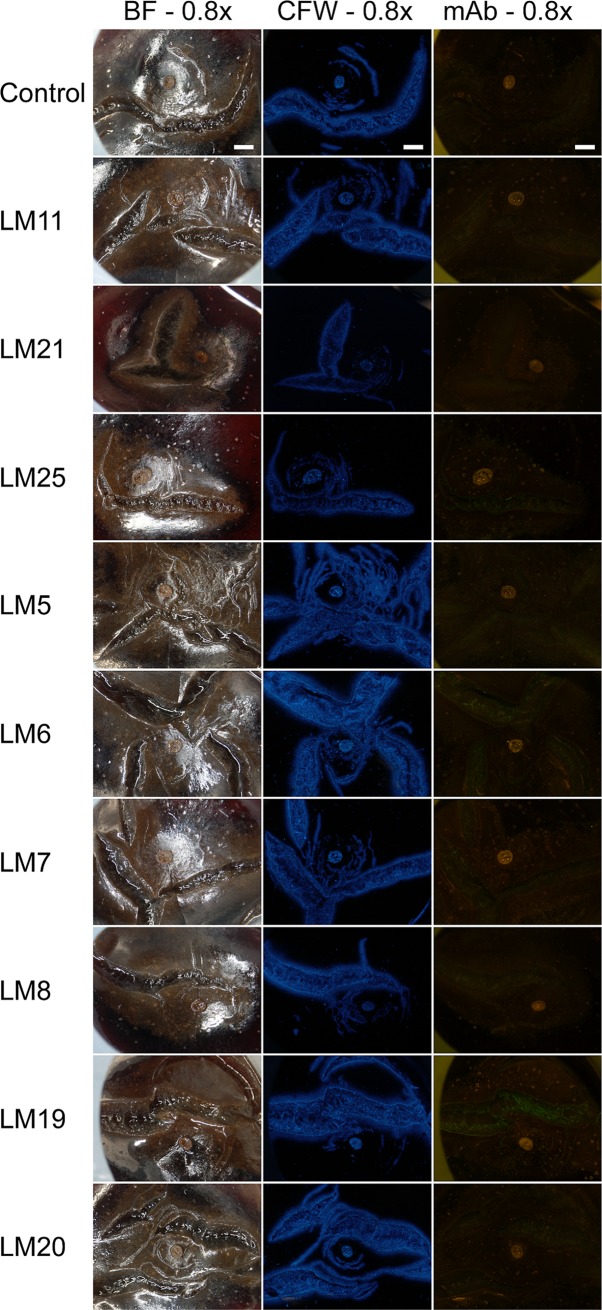
Composite of micrographs of ‘Burlat’ sweet cherry fruit that macrocracked in the stylar scar region during incubation in deionized water. Images were obtained following staining with calcofluor white or following binding of monoclonal antibodies (mAbs) against epitopes on cell walls exposed in the macrocrack surface. The mAbs reacting with epitopes of hemicelluloses were LM11 (anti-xylan/arabinoxylan), LM21(anti-mannan) and LM25 (anti-xyloglucan). The mAbs against pectin epitopes were LM5 (anti-galactan), LM6 (anti-arabinan), LM7 (anti-homogalacturonan), LM8 (anti-xylogalacturonan), LM19 (anti-homogalacturonan) and LM20 (anti-homogalacturonan). The first column of the composite was obtained under bright field illumination, the second column following calcofluor white staining (CFW) using UV light and the third column under fluorescent light following mAbs binding. All images 0.8x. Scale bar = 1 mm.

Micrographs reveal that sweet cherry suffered severe macrocracking in the stylar scar region and that both microcracks and macrocracks stained with calcofluor white. When treating macrocracks with mAbs, we observed no significant binding of the mAbs specific for hemicelluloses (LM11, LM21) but some weak binding with LM25 specific for xylo-glucans. Among the mAbs specific for pectins, the strongest binding was with LM19 specific for unesterified homogalacturonan. Other pectic epitopes were identified at markedly lower levels as indexed by very weak fluorescence. These included arabinans (LM6), xylo-galacturonans (LM8) and esterified homogalacturonans (LM20). Compared to unesterified homogalacturonan (LM19), esterified homogalacturonan was exposed on the macrocrack surface at low level (Fig 6).

**Fig 7:**
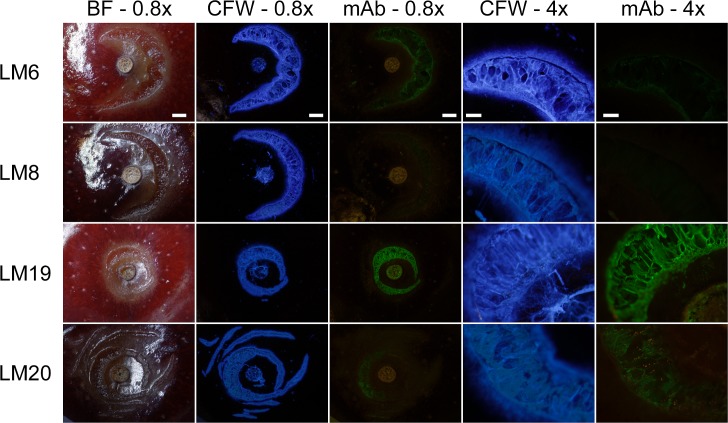
Composite of micrographs of ‘Samba’ sweet cherry fruit that had macrocracked in the stylar scar region during incubation in deionized water. Images were taken following staining with calcofluor white (CFW) or following binding of monoclonal antibodies (mAbs) against epitopes on cell wall surfaces exposed in macrocracks. The mAbs reacting with epitopes of hemicelluloses were LM6 (anti-xylan/arabinoxylan), LM8 (anti-xylogalacturonan), LM19 (anti-homogalacturonan), and LM20 (anti-homogalacturonan). Images were viewed under bright field illumination (BF), under UV (CFW) or under fluorescent light (mAb). Scale bars in the first, second and third columns = 1 mm, in the fourth and fifth columns = 0.2 mm.

Detailed investigations of macrocracks using the mAbs LM6, LM8, LM19 and LM20, all against pectins, in a different sweet cherry cultivar, revealed a similar pattern of binding to the surfaces of microcracks and macrocracks (Fig 7). At higher magnification, stretching of cells normal to a microcrack and the beginning of cell:cell separation along a developing macrocrack were detectable, following calcofluor white staining or following binding of LM19.

**Fig 8:**
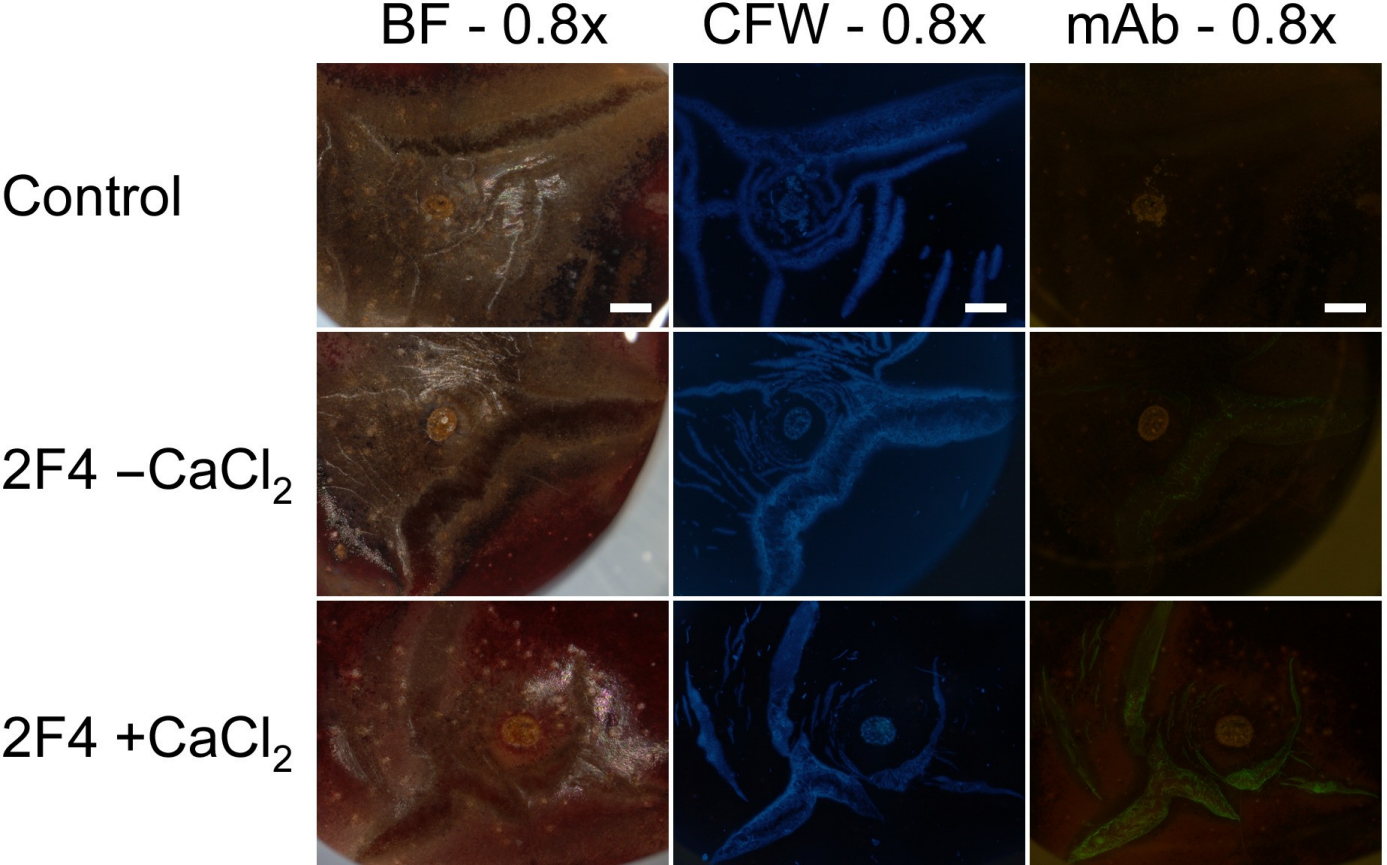
Composite of micrographs of ‘Burlat’ sweet cherry fruit that cracked in the stylar scar region during incubation in deionized water. A) Bright field image (BF). B) Same specimen as A, but macrocracks stained using calcofluor white (CFW) and viewed under UV light. Same specimen as A, but now treated with the monoclonal antibodies LM19 (anti-homogalacturonan) and viewed under fluorescent light. D) Detailed view of cracks labeled with LM19 showing crack network with a microcrack (‘zone II’) and a developing macrocrack (‘zone III’). Scale bars = 1 mm (A-C) or 100 μm (D). In the microcrack (zone II), the cuticle has fractured, but epidermal cells are largely intact. The mAb LM19 labeled the periclinal cell walls. In the macrocrack in zone III separation of epidermal cells along their anticlinal cell walls began near the tip. Separation proceeded along the macrocrack and gaping began (indicating release of stress and strain).

At times, microcracks and macrocracks branched or merged, particularly when cracking density was high, as was often the case in the stylar scar region (Fig 8A–8C). Nevertheless, the characteristic zoning (I, II and III) of macrocracking was still clearly discernible. Fig 8D shows a microcrack (zone II) that branched into a macrocrack (zone III). In the microcrack, the cuticle has fractured but epidermal cells are largely intact. Here, the mAb LM19 labeled the periclinal epidermal cell walls just beneath the ruptured cuticle. In the macrocrack (zone III), cell:cell separation of anticlinal epidermal walls began close to the macrocrack tip. Cell:cell separation proceeded along the macrocrack, with gaping indicating the release of stress and strain. Here, the mAb LM19 labeled the anticlinal cell walls at an intensity similar to the periclinal walls in zone II.

**Fig 9:**
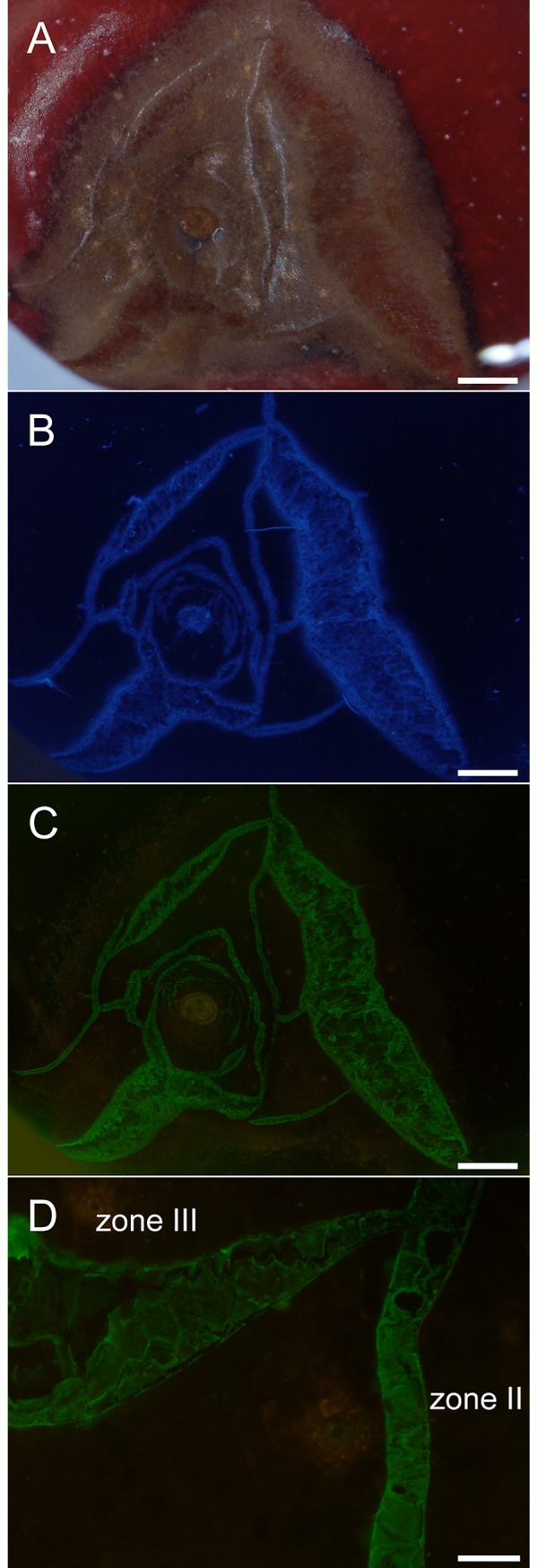
Composite of micrographs of ‘Burlat’ sweet cherry fruit that had cracked in the stylar scar region during incubation in deionized water. Images were taken following staining with calcofluor white (CFW) or following binding of the monoclonal antibody (mAb) 2F4 in the absence (2F4, -CaCl_2_) or presence of Ca (2F4, +CaCl_2_). The mAb 2F4 identifies dimeric associations of homogalacturonan chains with Ca^2+^. Images were viewed under bright field (BF), in UV (CFW) or in fluorescent light (mAb). All images at 0.8x. Scale bars = 1 mm.

Applying the mAb 2F4 specific for dimeric associations of homogalacturonans through calcium (Ca), revealed little binding to macrocracks indicating a low degree of Ca^2+^ cross linking. That Ca^2+^ binding sites were present is demonstrated by the stronger binding obtained when 2F4 was applied together with CaCl_2_ (Fig 9).

## Discussion

Our results indicate 1) that macroscopic cracking (macrocracking) of sweet cherry fruit resulted from increases in both macrocrack number and length, 2) that microcracking of the cuticle, the swelling of epidermal cell walls, the death of epidermal cells along a microcrack and the anticlinal separation of epidermal cells are all closely related, 3) that epidermal cells separated predominately along their anticlinal cell walls, and 4) that failure of cross linking in the middle lamella was responsible for the extension of a cuticular microcrack to a dermal macrocrack that extends deep into the flesh.

### Macrocracking, microcracking, swelling of epidermal cell wall, death of epidermal cells and separation of epidermal cells are all closely related

The time-lapse videos of fruit cracking reveal that macrocracking results from both the formation of new cracks and from crack extension–both measures increase during macrocracking. The macrocracks developed from microcracks by extension in the tangential direction (i.e. in the plane of the skin) and also in the radial direction (i.e. deep into the flesh) once the epidermal cells began to separate from one another. Cell separation was preceded by swelling of epidermal cell walls and by cell death. We obtained a close, positive, curvilinear relationship between the proportion of dead epidermal cells and the thickness of their anticlinal cell walls. As cell death progressed along a macrocrack, cell walls began to swell. That cell death occurred is concluded from the brownish coagulated cytoplasm and the loss of anthocyanin from the vacuole. Cell death is accompanied by a loss of turgor. Hence, the pressure on the cell wall decreases. This allows cell walls to swell. This conclusion is consistent with earlier findings where cell wall swelling was greatest for a cell wall between two non-living cells and least when both bordering cells were healthy and fully turgid [41]. The extent of cell wall swelling was intermediate for a cell bordering a healthy cell on one side and a non-living one on the other. Also, in mature fruit, cell wall swelling increased as the proportion of plasmolyzed cells increased, but not in immature fruit [41]. These observations indicate 1) that it is not cell death *per se* that is causal in the swelling of cell walls but the loss of turgor associated with cell death (or plasmolysis) [22] and 2) that chemical modifications of the cell walls, such as those during maturation [42], must precede cell wall swelling. In addition, cell death releases malic acid into the apoplast. Malic acid is a major osmolyte in sweet cherry that will tend to extract Ca^2+^ from the cell wall, thereby decreasing cross linking and exacerbating cell wall swelling [29]. From a physical point of view, cell turgor simply prevents water absorption and, hence, cell wall swelling as long as cell turgor exceeds the ‘swelling pressure’ of the cell wall matrix. Because turgor is very low in stage III sweet cherry [43, 44], the swelling pressure generated by the cell wall is expected to be even lower. Cell wall swelling is not unique to sweet cherry but occurs in fruit of many other species during normal ripening [24]. Cell wall swelling markedly decreases the fracture tension of the skin, hence, normal growth-induced skin tension [7] is enough to bring about its failure [22].

### Epidermal cells separate along their anticlinal walls indicating failure of the middle lamella

Skin failure is predominately schizogenous–i.e. cell:cell separation due to failure of the middle lamellae between the anticlinal walls of adjacent epidermal cells. Few cells fail lysigenously–i.e. rupture across the cell walls. This observation is consistent with results obtained in biaxial tensile tests using excised skins [22]. In these tests, an excised skin segment is pressurized from its inner surface and the extent of bulging quantified. Skin segments of mature fruit with swollen cell walls mostly failed schizogenously. Schizogenous failure is indicative of failure of the pectins of the middle lamella. This conclusion is supported by the binding of mAbs specific for epitopes of pectins. The strongest binding was obtained with LM19 and markedly with weaker LM20, LM6, LM8 and 2F4. These mAbs identify homogalacturonan domains (LM19, LM20, 2F4), xylogalacturonan (LM8) and arabinan domains (LM6) of pectic polysaccharides. It is worth noting that LM19 and LM20 are specific for unesterified and esterified homogalacturonans, respectively. Hence, the homogalacturonans exposed on the surfaces of an extending skin macrocrack are largely de-methylated, as would be expected for a mature, ripe fruit. During maturation and ripening, we would expect the intensity of labeling of homogalacturonans with LM19 to increase and that by LM20 to decrease. Furthermore, the weak binding of 2F4 (relative to the number of binding sites available) indicates a low level of crosslinking between pectins by Ca^2+^.

Failure of the pectin middle lamella is also consistent with the observation that the rate of strain affected the mode of failure of excised fruit skins [21]. The percentage of cells where fracture occurred schizogenously was higher at low strain rates, compared with at high rates [21]. Pectins exhibit viscoelasticity and time-dependent deformation (as also does the fruit skin) which are typical for viscoelastic materials [45].

From a practical point of view, schizogenous separation of skin cells, i.e. along the middle lamellae, offers the chance that macrocracking can be reduced by applications of Ca-salts. As shown by binding of 2F4 with simultaneous applications of Ca-salts, Ca^2+^ ions increase homogalacturonan cross linking [42, 46]. Increased cross linking also accounts for reduced macrocracking when Ca solutions are applied in the field during rain by overhead sprinklers [47] (for review see [48]). Similarly, a decrease in the amount of water uptake at 50% fruit cracking when fruit were incubated in solutions of CaCl_2_, FeCl_3_ and AlCl_3_ was reported [49]. Interestingly, the morphology of the cracks was also affected. Cracks were deep and gaping after incubation in water but less deep and less extensive when incubated in FeCl_3_. Both observations are easily accounted for in terms of cross linking of homogalacturonans by di- and trivalent ions [49]. Cross linking delays or stops the development of a microcrack into a macrocrack.

## Conclusion

**Fig 10:**
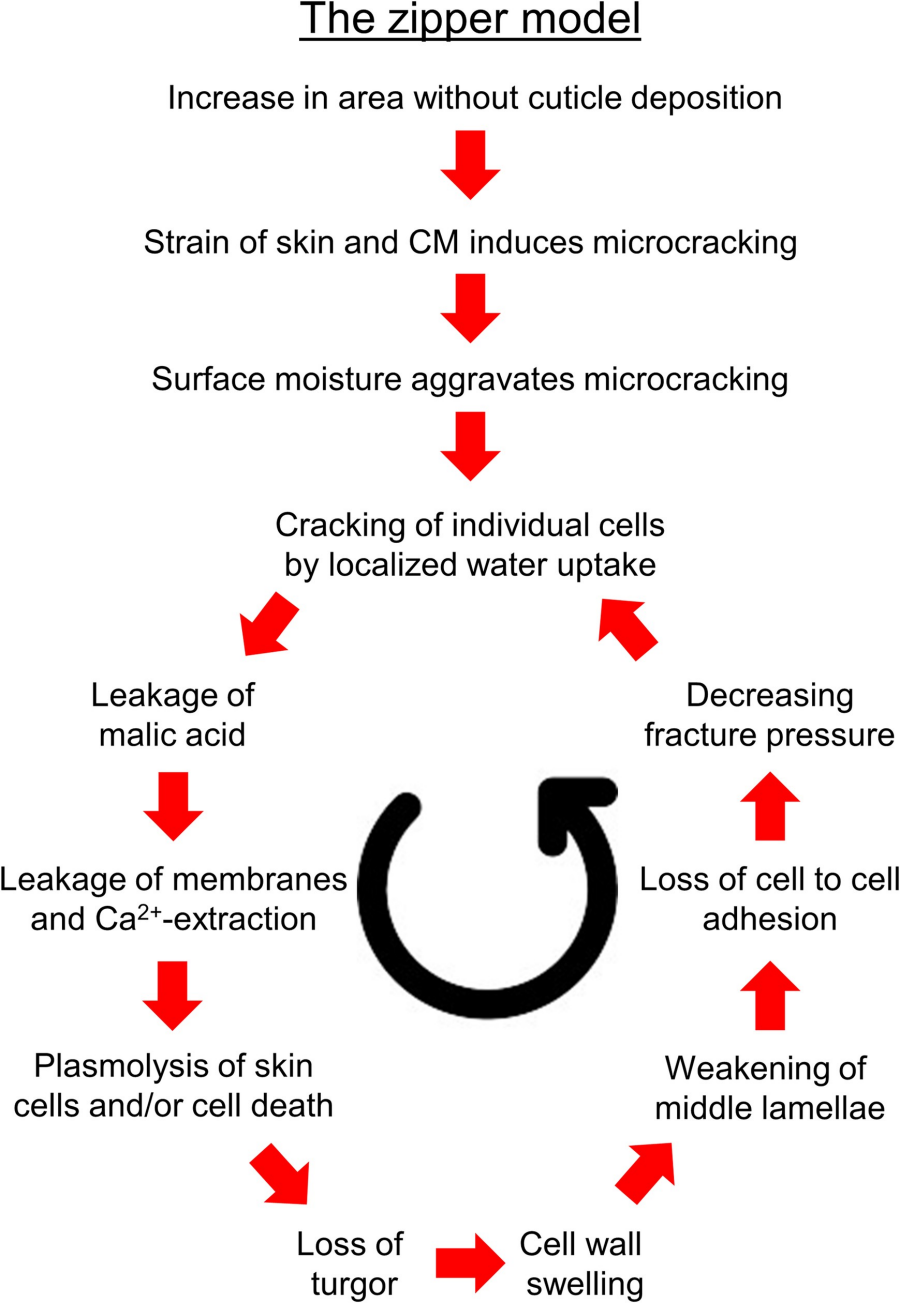
Sketch illustrating the sequence of events in macroscopic rain-cracking of sweet cherry. The increase in surface area in the absence of cuticle deposition [5] causes tension [7] and formation of microcracks [6]. Microcracking is aggravated by surface moisture [50]. Microcracks impair the cuticle’s barrier function [50] and focus water uptake in a particular region of the fruit surface [30]. Water penetration causes individual cells to burst, probably in the outer mesocarp where the osmotic potential is more negative than in the skin [41]. Malic acid leaks into the apoplast causing further leakage of membranes in surrounding cells and the extraction of Ca^2+^ from the cell wall [29]. Cells plasmolyse and collapse. Due to the loss of turgor, cell walls swell [22]. Swelling results in a weakening of the pectin middle lamella, the loss of cell to cell adhesion and decreased fracture pressure [22]. The cells separate along the middle lamella (this paper), the crack propagates as the skin “unzips” like a ladder in knitted fabric. This model is referred to as the zipper model [30].

Cuticular microcracks develop into skin macrocracks. This progression involves 1) cell death, 2) leakage of cell contents (esp. malate) and 3) cell wall swelling. These conclusions were obtained in experiments involving a range of different cultivars. There was no indication for cultivar specific interactions. Thus, the above conclusions are applicable to sweet cherry in general. The structural properties of sweet cherry skin are determined predominantly by the cellular layers (epidermis and hypodermis) while its barrier properties are determined predominantly by the cuticle. Microcracks impair the barrier properties. They result from strain of the cuticle due to a cessation of cuticle deposition during early fruit development and—possibly–from exposure of the strained cuticle to surface wetness (Fig 10). Microcracks focus water uptake in a particular region of the fruit surface. Due to the impaired barrier function of the cuticle, water penetrates–probably into the outer mesocarp where the osmotic potential is more negative than in the skin. Individual cells collapse. The liberation of malic acid following cell damage causes leakage of membranes of adjacent cells and extracts Ca^2+^ from the cell walls. Cell walls begin to swell. This weakens the skin locally, so it tends to unzip as the crack propagates–much like a ladder (run) in a knitted fabric. Cell wall swelling particularly weakens cell:cell adhesion causing dermal cells to separate schizogenously–i.e. neighbouring cells part from one another along their anticlinal middle lamellae. The schizogenous failure mode is indexed by the exposure of non-esterified homogalacturonans (LM19) on the fracture surfaces of a skin macrocrack. This sequence of events accounts for rain cracking in sweet cherry and will more than likely be found to occur also in other rain-susceptible fleshy fruit.

## Supporting information

S1 Dataset Raw data on crack formation and cell wall swelling that are displayed in figures and tables. (XLSX)
