# Correction: Crack initiation and propagation in sweet cherry skin: A simple chain reaction causes the crack to ‘run’

**DOI:** 10.1371/journal.pone.0247692

**Published:** 2021-02-19

**Authors:** Christine Schumann, Andreas Winkler, Martin Brüggenwirth, Kevin Köpcke, Moritz Knoche

The images for Figs 8 and 9 are incorrectly switched. The image that appears as Fig 8 should be Fig 9, and the image that appears as Fig 9 should be Fig 8. The figure captions appear in the correct order.

**Fig 8:**
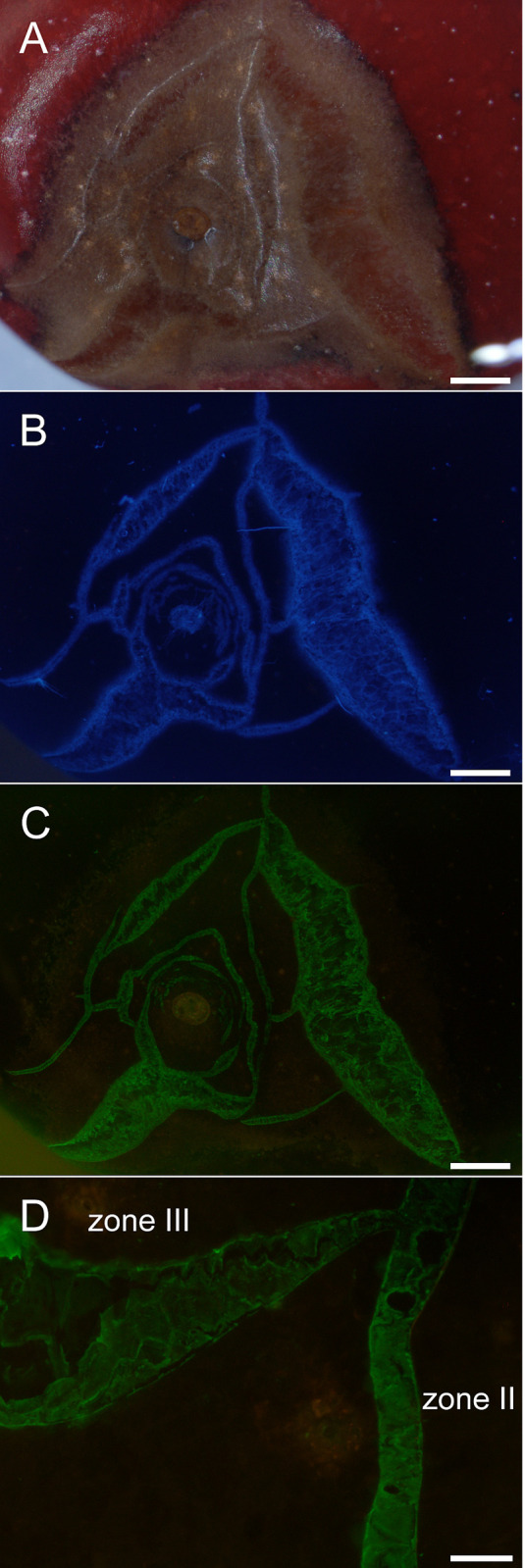
Composite of micrographs of ‘Burlat’ sweet cherry fruit that cracked in the stylar scar region during incubation in deionized water. A) Bright field image (BF). B) Same specimen as A, but macrocracks stained using calcofluor white (CFW) and viewed under UV light. Same specimen as A, but now treated with the monoclonal antibodies LM19 (anti-homogalacturonan) and viewed under fluorescent light. D) Detailed view of cracks labeled with LM19 showing crack network with a microcrack (‘zone II’) and a developing macrocrack (‘zone III’). Scale bars = 1 mm (A-C) or 100 μm (D). In the microcrack (zone II), the cuticle has fractured, but epidermal cells are largely intact. The mAb LM19 labeled the periclinal cell walls. In the macrocrack in zone III separation of epidermal cells along their anticlinal cell walls began near the tip. Separation proceeded along the macrocrack and gaping began (indicating release of stress and strain).

**Fig 9:**
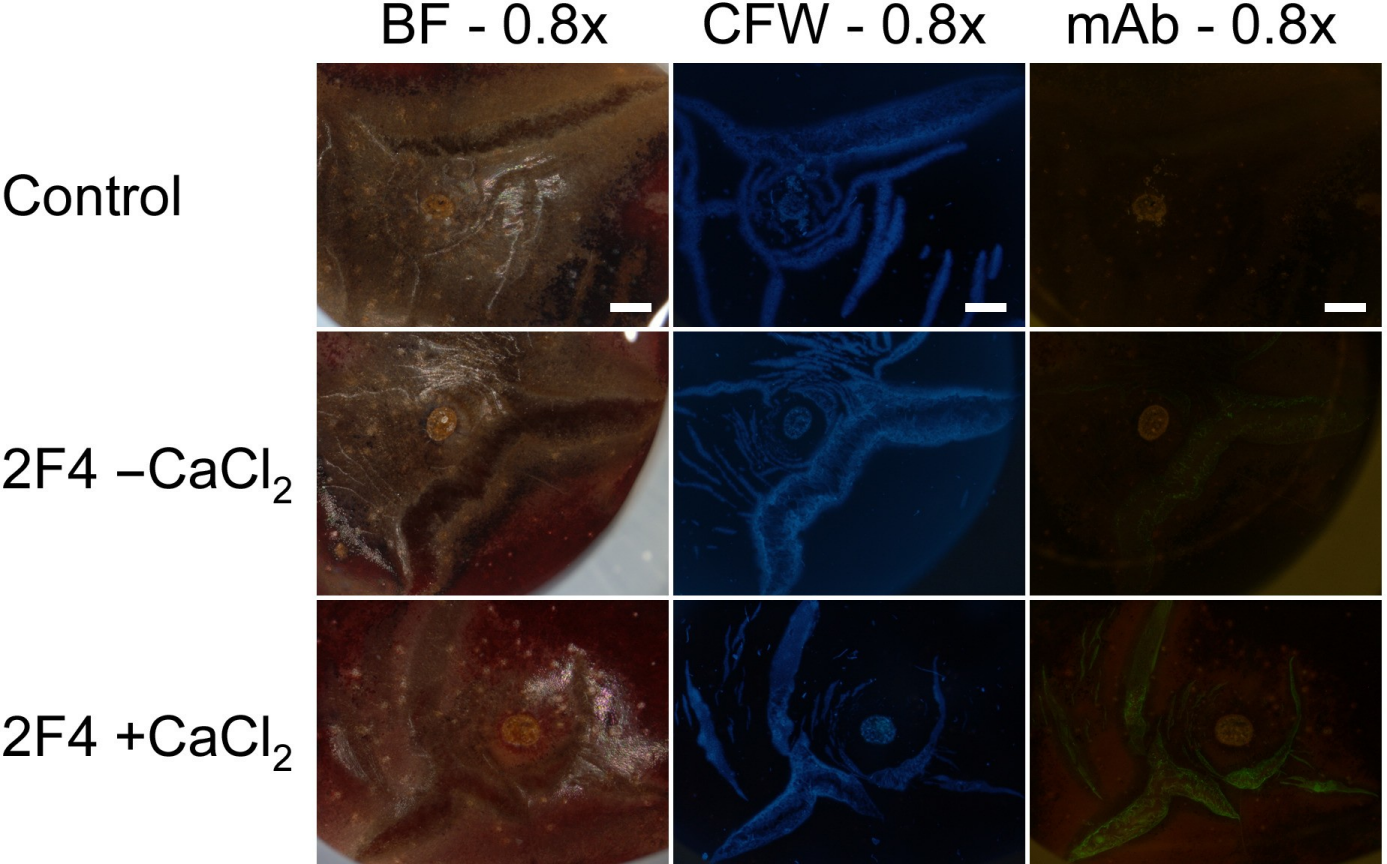
Composite of micrographs of ‘Burlat’ sweet cherry fruit that had cracked in the stylar scar region during incubation in deionized water. Images were taken following staining with calcofluor white (CFW) or following binding of the monoclonal antibody (mAb) 2F4 in the absence (2F4, -CaCl_2_) or presence of Ca (2F4, +CaCl_2_). The mAb 2F4 identifies dimeric associations of homogalacturonan chains with Ca^2+^. Images were viewed under bright field (BF), in UV (CFW) or in fluorescent light (mAb). All images at 0.8x. Scale bars = 1 mm.
